# Online level-2 perspective taking for newly learnt symbols

**DOI:** 10.1007/s10339-024-01244-7

**Published:** 2024-11-21

**Authors:** Réka Pető, Fruzsina Elekes, Ildikó Király

**Affiliations:** MTA–ELTE “Lendület” (Momentum) Social Minds Research Group, Institute of Psychology, ELTE Eötvös Loránd University, Izabella Street 46, Budapest, 1064 Hungary

**Keywords:** Perspective taking, Cultural symbols, Object appearance, Aspectual, Common ground

## Abstract

Humans demonstrate spontaneous sensitivity to other people’s perspectives on object identities in online tasks. Evidence shows that this not only involves representing the mere discrepancy between perspectives, but the content of such perspectives as well (level-2 perspective taking/L2PT). However, this evidence comes from studies using culturally grounded symbols which leaves open the possibility that having extensive, easily accessible background knowledge about an object is necessary for the L2PT effect. Experiment 1 tested this by comparing L2PT across two groups: one performing a verification task on Arabic numbers, and one on newly learnt symbol-label pairs. In both groups, half of the visual stimuli was symmetrical, while half was asymmetrical. In both cases, there was a joint condition: participants performed the task in parallel with a partner, observing stimuli from opposite angles, thus having conflicting interpretations for asymmetric characters. Furthermore, they also performed the verification task individually, while their partner had no visual access to the stimuli. We found an interference effect in both groups. However, while the effect was stable in the number group, it diminished over time in the symbol group. Experiments 2a and 2b demonstrated that the complexity of the recently learnt symbols has an influence on spontaneous L2PT: the same procedure with more complex symbols did not elicit any interference effect. Our results show that online L2PT is not limited to objects that participants have proficiency in identifying. Nevertheless, the L2PT effect seems to diminish when participants have to process increasingly complex novel symbols.

## Introduction

How fellow individuals conceive of the visual world—and thus, the epistemic states they acquire—is of utter importance to their interactive partners. However, this is dependent on the perspectives they take. The visual perspective of another person can be represented at different levels of complexity: it ranges from simply tracing one’s line of sight, a geometrical relation between the eyes and an object, to determine visual access (level-1 perspective taking), to representing how something appears from another person’s point of view, which requires dealing with alternative possible representations of reality (level-2 perspective taking, L2PT, Flavell, Everett, Croft, & Flavell et al. 1981). At its richest, L2PT requires handling the perspective problem which involves understanding that seemingly irreconcilable modes of presentation, for instance, conflicting perceived object identities, may all describe the referent truthfully under the assumption that representations are formed in different perspectives (Perner et al. 2003).

In this current paper, we will review evidence about humans’ ability and willingness to spontaneously engage in level-2 visual perspective taking, focusing on the representation of alternative object identities or appearances.^1^ We will pay particular attention to what conclusions the findings from available paradigms allow in terms of representational complexity of this skill, and we will point out a methodological feature of recent investigations that constrains our understanding of spontaneous L2PT. Namely, that the vast majority of studies use culturally grounded, alphanumeric symbols as stimuli. Adult participants have automatized knowledge of these symbols and assume that other people know and understand them the same way they do. Whether this deep-rooted knowledge of object identities is a precondition of PT has not been tested yet. We will present a study that addresses this question by using recently learnt symbols as stimuli, instead of well-known alphanumeric ones.

One approach to examining whether humans engage in level-2 perspective taking is testing participants’ spontaneous verbal reports of the identity of ambiguous stimuli. A typical stimulus includes the number character 6/9—the identity of which depends on spatial orientation—viewed by a fellow agent from an opposite angle. Participants are asked to report the identity of the number amongst unrelated filler questions (Quesque et al. 2018; Zhao et al. 2015). It has been found that a substantial proportion of participants spontaneously adopt the perspective of the agent with regards to the object—in the example, the symbol of 6/9 -, and this tendency is further enhanced if the agent exerts attention or behavioural intentions toward the object (Zhao et al. 2015; Zhao et al. 2016a).

However, similarly to other cultural artefacts such as tools (Defeyeter and German, 2003; Kelemen and Carey 2007), symbols also have an intended meaning (DeLoache 1995), that humans are sensitive to from an early age (Apperly et al. 2004). The intended nature of symbols may lead participants to link the orientation of the object to the agent present *within* the physical representation (pictorial stimulus), thereby increasing the spontaneous PT effect. Furthermore, since the intended meaning of a symbol is independent of the agent’s current visual access, the nature of cultural symbols may explain why participants take the alternative perspective even when the agent’s vision is disrupted by a blindfold, or when only a chair is present to indicate the alternative viewpoint (Quesque, et al. 2018). These findings may involve perspective taking in the spatial, navigational sense (Ward et al. 2019b), representing how the object looks from the “right side up”, but not in the social sense, representing another agent’s *mental representation*.

Closer to the social focus of the present work are studies in which participants are *instructed* to rely on their own or the other’s perspective when judging the identity of the number and it is assessed whether their responses (in terms of response efficiency, RT-s, or error rates) are nevertheless influenced by the alternative perspective. This is usually tested with the so-called number verification paradigm (Surtees et al. 2012). In this task, the participant observes a room with a table next to a wall and an avatar behind the table. On each trial, a number (0,8,6,9) is presented either lying on the table or hanging on the wall. The numbers on the wall look similar to both the avatar and the participant, while the numbers on the table look different to the two agents if the number is asymmetrical (6,9). The participant’s task is to verify the identity of the number either from their own or the avatar’s perspective. When instructed to respond from their perspective, the participants’ response efficiency decreases for asymmetric numbers presented on the table. This effect, termed altercentric interference, is assumed to reflect the spontaneous, online computation of the avatar’s perspective and has been documented by multiple independent investigations with slight variations of the task (Elekes et al. 2016; Surtees et al. 2016a, b; Surtees et al. 2016a, b).

However, it is still debated how much the online PT mechanism can capture the representational complexity of L2PT. Firstly, does the mechanism reflected in the altercentric interference entail representing other humans’ *mental states*, or merely a possible alternative reading of the target? Secondly, does the interference effect originate from representing the specific *perspective content* held by the partner or simply that there is a difference in perspective?

On the first point, there is some evidence from the number verification paradigm which shows that the partner’s intentional relation with the stimuli modulates the interference effect. This suggests that online PT indeed maps out mental representations (Elekes et al. 2016). The authors found that the partner’s presence only led to online perspective interference when the partner was known to pay attention to, and represent the stimulus in terms of the magnitude the digit referred to rather than its surface feature, and presentation colour. However, such selectivity was found absent in an experiment where participants were instructed to make eye contact before each trial (Surtees et al. 2016a, b), indicating that the gross sociality of the task (repeated cues to joint attention) may overwrite this nuanced effect. Whether (or else, under what conditions) perspective interference reflects an understanding of the other’s mental realm needs further testing in light of work showing that the mere presence of another individual can elicit such effects (Kuhn et al. 2018).

Regarding the question of representing perspective content, accumulating evidence indicates that the PT ability reflected in online measures goes beyond tracking *differences*, and constitutes representing the alternative object *appearance* or identity itself (Freundlieb et al. 2018; Ward et al. 2019a, b; Yuan et al. 2017). In Yuan et al. (2017) study, participants judged the motion direction of a set of dots moving left or right with varying motion coherence *after* viewing a motion adaptor scene with vertically moving stimuli. The adaptor scene was also witnessed by an avatar situated left to the participant (who thereby was adapted to a left/right motion) or was present with eyes closed. When the avatar’s eyes were open, participants were found to take the avatar’s perspective and adapt to a left/right motion, lessening their sensitivity to that motion direction and creating a motion aftereffect at the test.

Further evidence for computing object appearance online comes from Freundlieb et al. (2018) experiments. In this study, participants saw words from two semantic categories on a screen lying flat on a table in the presence of a confederate who sat 90° from the participant. The words were oriented vertically from the participant’s perspective and were either right side up (easy to read) or upside down (hard to read) *for the confederate*. In a semantic go/no-go task, Freundlieb and colleagues reported quicker processing of the words when they faced the confederate, suggesting that in addition to the existence of a difference between perspectives, the content of the alternative perspective was also computed. Finally, in a recent study (Ward et al. 2019a, b) participants performed a computerised mental rotation task on alphanumeric characters. In some trials, the stimulus also included a human figure looking at the character, sitting on the left or right of the participant. As typical in a mental rotation task, the authors found increasing RT-s as the degree of rotation increased. However, decisions were *facilitated* when the character was rotated towards the fellow agent, suggesting that participants could make use of the avatar’s perspective when the character appeared upright to him (for a similar finding, see Böckler et al. 2011).

To conclude, evidence from multiple paradigms suggests that human adults can reach alternative object appearances (mode of presentation) spontaneously even when that requires quick and repeated updating of the content of the partner’s perspective.

Note, however, that except Yuan et al. (2017) study on the motion aftereffect, evidence for online level-2 PT, comes from paradigms that asked people to judge the identity of well-known symbols, such as numbers or letters. The meaning of these culturally defined symbols is stored in long-term memory and can be accessed relatively automatically from around the age of 6–9 years (Dencla and Rudel 1974; van Galen and Reitsma 2008), continuing into adulthood. It has been proposed that excessive background knowledge about the target symbols could contribute to the fluency with which the partner’s perspective is updated in these tasks (Elekes et al. 2016; Westra 2017). It is, therefore, an open question whether online L2PT would generalise to objects with which people are less familiar, and which lack such deep-rooted and widely shared meaning.

This question gains further importance in light of recent research targeting how the amount of available cognitive resources affects implicit level-2 PT. The process of perspective-taking requires a high load on cognitive capacity (calculating a person’s own and the other’s possible perspective contents; understanding the person’s own and the other’s goals…etc.), therefore, it is conceivable that cognitive workload influences level-2 PT. We know of two studies that aimed to directly explore how taxing the cognitive system would affect level-2 PT. Deliens et al. (2018) reported that total sleep deprivation (a manipulation that is known to affect executive functioning, and mood) does not modulate L2PT interference. Similarly, according to Todd et al. (2019), participants continued to show altercentric interference from the avatar’s level-2 perspective when processing opportunities were constrained by time pressure, although process dissociation analysis revealed that perspective calculation decreased under such conditions. On a more indirect note, there is evidence that 8—to 9.5-year-old children show similar level-2 perspective interference as adults despite being generally less efficient in their number verification decisions (Elekes et al. 2017). This implies that a lack of proficiency with stimulus identification itself and an enhanced cognitive demand a task may impose does not necessarily diminish PT.

However, we do not know of any study that investigated the influence of cognitive capacity on spontaneous level-2 PT by taxing cognitive capacity with stimuli that varied in complexity. We hypothesise that certain attributes of the stimuli have a significant influence on L2PT. When participants encounter stimuli that they recognize spontaneously, they can fully devote their cognitive resources to the perspective-taking task, which may lead to increased attention to the other person's point of view. If, on the other hand, part of the cognitive capacity is used for other parallel tasks (such as recognizing newly learnt symbols), this can lead to a lower capacity for spontaneous perspective-taking. Based on this and previous findings with children as symbol novices, we predicted that spontaneous level-2 perspective interference would extend to judgments of recently learnt visual stimuli in adults, but only if the newly learnt stimuli did not exceed their cognitive capacity. Therefore, we planned a study in which we could use symbols of varying complexity to investigate whether the extent to which cognitive capacity is used for a parallel task affects L2PT.

Consequently, the aim of the current study is twofold. First, Experiment 1 was created to test in a controlled manner, whether altercentric perspective interference is constrained to highly familiar alphanumeric stimuli or is also evident regarding symbols that adult participants have only encountered within the context of the experiment. In addition, Experiment 2 was conducted to shed light on the possibility that the complexity of the stimuli also has an impact on the process of spontaneous level-2 PT and may weaken or even diminish in the case of a cognitively more demanding setup. We believe that our results could contribute to the extant literature by demonstrating that online PT is not limited to symbols that require deep-rooted knowledge and that the cognitive capacities involved in the process of retrieving newly learnt symbols from the memory system and the computation of the recently learnt symbols alternative object identities may surpass the capacities of the online PT mechanism (Apperly and Butterfill 2009; Edwards and Low 2019).

Furthermore, such a finding would also indicate that Theory of Mind (ToM) can spontaneously recruit information stored in long-term memory (whenever there is such) to facilitate its functioning when the computation itself is too demanding to be achieved online and would thereby add to an existing literature on the interaction between ToM and other cognitive processes (Conway et al. 2019; Westra 2019).

## Experiment 1

In Experiment 1, participants were first presented with 4 novel visual symbols (2 symmetric, 2 asymmetric) and a label for each symbol (pseudo-words in the participants’ native language). The two asymmetric characters could be rotated into each other, hence different labels belonged to the same physical object depending on stimulus orientation, similarly to the 6/9 character. The symbols used in Experiment 1 were asymmetric on the horizontal axis, but not the vertical axis. Note, while participants were instructed to learn the labels for the novel symbols, the meaning—the referents—of the symbols remained opaque. Despite this, the symbol–label mapping could represent the initial step of how culturally constituted codes are acquired.

The experiment started with an about 15-min-long practice phase, which enabled participants to learn the new symbol-label pairs. Participants took part in this training session individually. After the brief individual practice phase, participants took part in a verification task with their partner. Participants sat on opposite ends of a table, observing the stimuli—presented on a flat screen laid on the table—from opposite perspectives in the joint phase, and individually in the individual phase.

While the training phase was similar for all the participants, the test phase was different. Half of the participant pairs saw number stimuli on the screen (number verification group), while the other group saw the newly learnt symbol-label pairs (symbol verification group). We compared the performance of these two groups of participants. We predicted that participants’ verification judgments would be less efficient for jointly observed asymmetric stimuli (i.e. altercentric interference) regardless of the type of stimulus—implying that the partner’s alternative representation of the perspective dependent symbols was tracked.

### Methods

**Participants.** A total of 44 adult participants were recruited and paired randomly from a university pool, as part of a course (titled: Participation in Psychological Experiments). Participant pairs took part in either the symbol verification (SV) or the number verification (NV) group (n = 22 per group). The experiment was approved by the ethical committee of our university.

**Materials.** Visual stimuli were created in Adobe Illustrator (see Table 1). The digital numbers and symbols were 11.5 cm in height, and 5.2 or 6.2 cm in width on a 21.5 in. flat monitor.

**Table 1:** Stimuli (symmetric and asymmetric) used in the number verification and symbol verification groups in Experiment 1 and 2

	Symmetric stimuli		Asymmetric stimuli	
Number (Exp1)				
*Newly learnt symbols*				
Symbol (Exp1)				
Symbol (Exp.2 a & b)				

In both groups, two of the symbols were symmetric, yielding the same percept if observed from opposing visual perspectives. The other two symbols were the two orientations of the same, asymmetric object and could be rotated into each other. In this experiment, the two asymmetric symbols could be transformed into one another with a 180-degree rotation, as they were vertically symmetrical. The participant only needed to vertically mirror the symbol to view the perspective of the person sitting across from them.

Four pseudo-words, in the participants’ native language (“róba”, “irim”, “opaj”, “kavu”) and four number labels (“nulla”, “nyolc”, “hat”, “kilenc”) were recorded by a female voice in a neutral, descending intonation used in the SV and NV groups, respectively. All of the pseudo words are borrowed from the Hungarian Pseudo-word Repetition Test (Racsmány et al. 2005). They are all words which consist of two syllables, follow the morphology of the language and thus, are supposed to be similar to the extent they tax working memory load.

**Procedure.** Participants were introduced to each other upon arrival and filled in an informed consent form. The experiment was presented in PsychoPy 3.0.5 using Python 2.7.15.

***Individual symbol learning and practice.*** To equate the length of the study, participants in both groups took part in a symbol learning and practice phase. Participants conducted the individual learning phase at two separated locations of the same testing room. They were told to memorise the symbol-label pairs and then to practice these pairings as follows. They first saw the four symbols on the screen side by side for 45 s with one of the labels below each symbol. The symbol-label pairs were randomised across pairs. After that, each symbol-label pair was presented one by one in the centre of the screen for 20 s each. The practice phase consisted of 4 mini-blocks of 20 trials with a fixed 30-s-long break between the blocks. In each practice trial, participants saw a symbol at the centre of the screen along with the four labels listed below it horizontally. Their task was to choose the label that matches the symbol by pressing the corresponding key (t, z, u, i—leftmost key referring to the label on the far left etc.). The label-key mappings were determined randomly and were re-randomized for each block. The key mappings for the subsequent block were presented during the break to let participants familiarise themselves with it.

Note, we varied the labels for the different symbols in a counterbalanced manner: e.g. for some pairs of participant ‘irim’ and ‘kavu’ were the labels for the symmetric symbols, while ‘róba’ and ‘opaj’ were the symmetric ones, for other pairs, this pattern was the opposite or mixed. This allows us to claim that specific symbol-label matches could not be easier than others.

*Common ground check.* When both participants finished the practice phase, the experimenter placed 4 cards in front of them, each depicting one of the four labels. She told them that she would place each symbol (printed on cardboard sheets) on the table, one at a time, and that they would have to take turns choosing the matching label for them. When the pairings were reconstructed this way, the experimenter asked the participants if they both agreed that all four pairs were correct. All participant pairs could accurately recreate the pairs. Note, however, that it is possible that the mental representations of the stimuli varied among participants. During the task, one participant might have internally referred to one of the symbols as ‘arrow,’ while to the other, as ‘a fish.’ However, since these terms were not spoken out loud or discussed, they could not have influenced the other participant’s decision-making. In this experiment, establishing common ground meant ensuring that participants were aware they had learned the same symbol-label pairs. This check was intended to confirm that both participants understood that the four pseudo-words represented the same symbols. Consequently, when they see a symbol, both participants would have its mental representation, which could either match or differ depending on whether the symbol was symmetrical or asymmetrical.

***Perspective taking test.*** Before the test, participants were told that they would take part in the main experiment together and that their task would either concern the symbol-label pairs they had just learnt (SV group) or some number characters unrelated to the symbols they had learnt (NV group). Furthermore, the instruction declared that in some parts, they would be doing the task in parallel, while in other phases, only one of them would perform it, while their partner would sit passively with their back to the set-up. Participants were informed that they would hear a label and see a picture (either a symbol or a number), and their task would be to decide if what they saw matched the label that they heard.

**Fig. 1:**
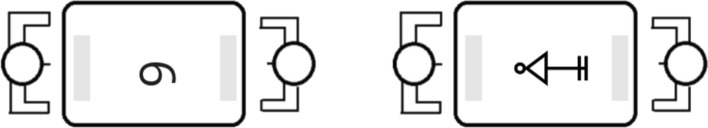
During the task participants sat facing each other at opposite ends of a table. Between them was the screen on which the symbols were displayed

During the test, participants were seated on the opposing sides of a short, 138 cm long table (see Fig. 1). A flat screen was placed between them, lying on its back on the table, on which the visual stimuli were displayed. A pair of loudspeakers was placed next to the participants through which the labels were presented in the auditory modality. Each trial started by a fixation cross being presented in the centre for 600 ms. After that, a label was presented through the loudspeakers followed by the visual stimulus with 200 ms stimulus onset asynchrony (SOA). SOA was set in a way that the first syllable of the label was already uttered when the visual image appeared. As the first syllable was definitive of the label, information necessary for the decision were presented approximately at the same time in the two modalities. The visual stimulus was shown on the screen until the active participant gave a response in the individual conditions or until both participants responded in the joint conditions. Visual stimuli were congruent (from the participant perspective) with the audio stimuli on half of the trials and non-congruent in the other half. In this way, trials were either congruent or incongruent for each participant. Congruent condition means that the label was in line with the visually seen symbol for the participant, while incongruent meant it did not match from their point of view. This allowed the opportunity to measure the difference between matching and mismatching perspectives.

Participants made their responses on Cedrus type response boxes, using their left and right hands. They were instructed to press the green button if the label matched the symbol on the screen and the red button when it did not. Key-mappings (left/right) were balanced within pairs. Cardboard boxes covered participants’ hands from sight.

The procedure started by an individual practice phase including 16 trials for both participants. The main experiment was divided into 2 halves. Each half effectively included a mini-experiment, consisting of both an individual phase for P1 and P2, and a joint phase. Each test phase consisted of 128 trials, half of which depicted a symmetric and half an asymmetric stimulus. In 50% of all trials, the picture-label pair was a matching (congruent, “yes” trials), while the other 50% included a non-matching pair (incongruent, “no” trials). Each phase was divided into two blocks of equal length, separated by a 30 s break. In the individual phase, the passive participant turned 180°, to face away from the stimuli, while in the joint phase, both participants sat facing the screen and both performed the verification task in parallel. The order of phases was one of the following two possibilities: Individual (P1)—Individual (P2)—Joint (P1 & P2)—break—Individual (P1)—Individual (P2)—Joint (P1 & P2); or Joint (P1 & P2)—Individual (P1)—Individual (P2)—break—Joint (P1 & P2)–Individual (P1)—Individual (P2).

After finishing the perspective taking test phase, participants were debriefed, and the experimenter answered their inquiries.

**Data cleaning.** The efficiency score was selected to be our main dependent measure, which is a frequently used metric in research on implicit perspective-taking (see also Elekes et al. 2017; Deliens et al. 2018). We calculated the efficiency score (ES) by dividing the average response times (RTs) by the percentage of correct trials (Townsend and Ashby 1978). This way, higher ES values signify more challenging decision-making processes. This measure helps to account for varying speed-accuracy trade-offs, which is particularly useful given the potential differences in strategies between participants, and across experimental groups. Having just acquired the symbol-label pairs, the speed-accuracy trade-off was probably larger in the SV group than in the NV group. By combining these two measures into a single value, the efficiency score can show a more complete picture.

We first performed the outlier rejection on the RT measure. Initial inspection of the descriptive statistical data revealed that RT-s and SD-s were reduced dramatically from the first to the second half of the experiment. Therefore, we computed the means and standard deviations separately for the first and second half of the experiment and used these to determine outlier data points in each half. Trials deviating by more than 2 SD-s from the mean RT were removed from the data. In the symbol group, 3.28% and 3.87% of the trials were excluded as outliers in the first and second half of the experiment, respectively (total: 3.57%). At the same time, 4.14% and 4.2% of all data points were identified as outliers in the first and second half of the experiment in the number group (total: 4.17%).

We then calculated the mean percentage of accurately answered trials (maximum provided by trial number after outlier rejection). Finally, the efficiency score was computed (as described above). Data was analysed in SPSS software version 25. All data subjected to statistical analysis is available at: https://osf.io/kb9qc/?view_only=fef32cc58a9b46b5993b657b408b54d7

### Results

**Symbol practice.** Participants’ overall accuracy in the symbol learning phase was 96.99% (SD = 3.26%) and 96.08% (SD = 2.82%) in the NV and SV groups, respectively. A mixed measures ANOVA with Symmetry as within-subject factor and Group as between-subjects factor revealed no significant differences between groups (*p* = 0.433), symmetry (*p* = 0.344) or their interaction (*p* = 0.587).

**Verification task.** We employed a 2 × 2 × 2 × 2 × 2 repeated measures ANOVA with Time (1st, 2nd half), Jointness (individual, joint), and Symmetry (symmetric, asymmetric) and Congruence (congruent, incongruent) as within subject factors and Group (NV, SV) as a between-subjects measure.

Our main focus was on the jointness x symmetry interaction. We expected higher efficiency scores in joint compared to individual participation for asymmetric but not for symmetric numbers. This pattern of data would indicate that the other’s perspective had been represented and causes interference when conflicting with the self-perspective.

Descriptive statistical data are presented in Table 2. All five factors had a main effect on efficiency scores: Time, *F* (1, 42) = 46.058, *p* < 0.0001, η_p_^2^ = 0.523, Jointness, *F* (1, 42) = 8.277, *p* = 0.006, η_p_^2^ = 0.165, Symmetry, *F* (1, 42) = 14.832, *p* < 0.0001, η_p_^2^ = 0.261, Congruence, *F* (1, 42) = 102.550, *p* < 0.0001, η_p_^2^ = 0.709, and Group, *F* (1, 42) = 20.164, *p* < 0.0001, η_p_^2^ = 0.324. Specifically, participants’ decisions got more efficient by the second half of trials, their responses were more efficient for symmetric than asymmetric symbols, in the individual over the joint task context and for congruent as opposed to incongruent trials. Finally, participants exerted more efficient decisions in the NV group. Group was also found to modulate the effect of both Time and Symmetry as reflected by 2-way interactions, Time x Group, *F* (1, 42) = 8.811, *p* = 0.005, η_p_^2^ = 0.173, and Symmetry x Group, *F* (1, 42) = 12.365, *p* = 0.001, η_p_^2^ = 0.227. Both the effect of symmetry and the effect of time was more robust in the SV group. We also found an interaction between Time and Jointness, *F* (1, 42) = 12.339, *p* = 0.001, η_p_^2^ = 0.227, where the deteriorating effect of joint task performance was found to decrease over time. Time also interacted with Congruence, *F* (1, 42) = 6.966, *p* = 0.012, η_p_^2^ = 0.142, and this was modulated by a three-way interaction with Group, *F* (1, 42) = 5.866, *p* = 0.020, η_p_^2^ = 0.123. The effect of Congruence decreased over time, and this was particularly so in the SV group (Table 3).

**Table 2:** Mean efficiency scores with standard deviations in parentheses in Experiment 1 and 2 as a function of Time, Jointness, Symmetry and Congruence

Time	Jointn	Symm	Cong	Experiment 1		Experiment 2	
				NV	SV	a	b
1st half	Ind	Symm	C^a^	6.602 (0.998)	7.866 (1.183)	7.746 (1.659)	7.987 (1.481)
			IC	7.239 (1.312)	8.897 (1.650)	8.64 (1.584)	8.753 (1.363)
		Asym	C	6.59 (1.098)	8.591 (1.715)	8.817 (1.773)	8.722 (1.781)
			IC	7.217 (1.241)	9.272 (1.554)	8.691 (1.770)	9.466 (2.057)
	Joint	Symm	C	7.112 (1.090)	8.051 (1.652)	8.033 (1.880)	7.953 (1.174)
			IC	7.600 (1.539)	9.240 (2.034)	8.96 (1.95)	8.848 (1.357)
		Asym	C	7.154 (1.097)	9.352 (2.504)	9.255 (2.034)	8.760 (1.590)
			IC	7.791 (1.374)	10.408 (2.618)	9.199 (2.026)	9.729 (2.229)
2nd half	Ind	Symm	C	6.306 (0.967)	6.968 (0.758)	7.062 (1.042)	7.165 (1.030)
			IC	6.925 (1.310)	7.584 (0.828)	7.767 (0.944)	8.013 (1.009)
		Asym	C	6.264 (0.7)	7.496 (1.051)	7.827 (1.514)	7.628 (1.201)
			IC	6.827 (1.166)	8.176 (0.883)	7.818 (1.367)	8.444 (1.620)
	Joint	Symm	C	6.41 (0.793)	7.037 (0.889)	6.979 (0.831)	7.162 (1.032)
			IC	6.82 (1.08)	7.62 (0.931)	7.564 (0.640)	7.873 (0.952)
		Asym	C	6.346 (0.787)	7.501 (0.919)	7.916 (1.551)	7.633 (1.099)
			IC	7.086 (0.982)	8.259 (1.046)	7.631 (0.921)	8.436 (1.951)

^a^C, congruent (visual and auditory information match); IC, incongruent (visual and auditory information do not match)

**Table 3:**
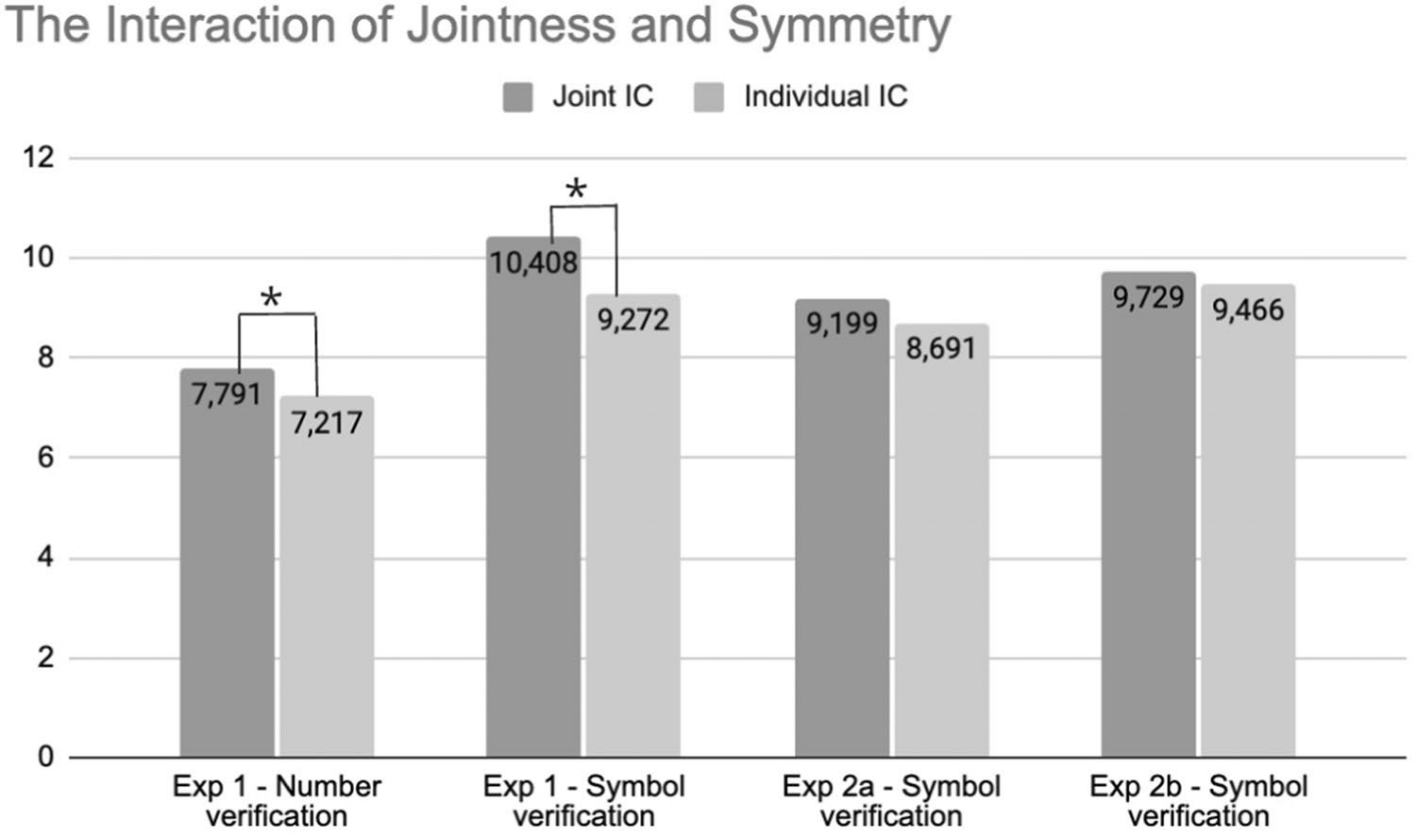
Depicts main results of the differences between joint and individual conditions in the case of asymmetric symbols in the first half of Experiment 1 and 2

The expected Jointness x Symmetry interaction was found significant, *F* (1, 42) = 9.409, *p* = 0.004, η_p_^2^ = 0.183, but it was modulated by a three-way interaction with Time, *F* (1, 42) = 6.537, *p* = 0.014, η_p_^2^ = 0.135, which shows that the perspective interference effect was more pronounced in the first than the second half of the experiment. Finally, we found a Time x Jointness x Symmetry x Group interaction, *F* (1, 42) = 8.1071, *p* = 0.007, η_p_^2^ = 0.162. This indicates that the way Time affects the targeted Jointness x Symmetry interaction is modulated by Group as well. Therefore, to test how Time affected the perspective interference effect in each group, we conducted separate 2 (Time: 1st, 2nd half) × 2 (Jointness: individual, joint) × 2 (Symmetry: symmetric, asymmetric) repeated measures ANOVA-s in both groups.

*Number verification group.* The ANOVA yielded the significant main effects of Jointness, *F* (1, 21) = 13.270, *p* = 0.002, η_p_^2^ = 0.387, Time, *F* (1, 21) = 19.080, *p* < 0.0001, η_p_^2^ = 0.476, and Congruence, *F* (1, 21) = 26.563, *p* < 0.0001, η_p_^2^ = 0.558. The interaction of Time and Jointness was also significant, *F* (1, 21) = 11.337, *p* = 0.003, η_p_^2^ = 0.351: The deteriorating effect of Jointness decreased over time. The Jointness x Symmetry interaction was significant, showing decreased efficiency for asymmetric stimuli in the joint phase compared to the individual, *F* (1, 21) = 6.673, *p* = 0.017, η_p_^2^ = 0.241. The expected interaction was not qualified further by Time, *F* (1, 21) = 0.124, *p* = 0.728, η_p_^2^ = 0.006, or Congruence, *F* (1, 21) = 2.208, *p* = 0.152, η_p_^2^ = 0.095.

*Symbol verification group.* The ANOVA revealed that Time, *F* (1, 21) = 29.407, *p* < 0.0001, η_p_^2^ = 0.583, Symmetry, *F* (1, 21) = 14.746, *p* = 0.001, η_p_^2^ = 0.413, and Congruence, *F* (1, 21) = 106.207, *p* < 0.0001, η_p_^2^ = 0.835, had significant main effects of efficiency scores. We also found an interaction between Time and Jointness, *F* (1, 21) = 5.048, *p* = 0.036, η_p_^2^ = 0.194, and between Time and Congruence, *F* (1, 21) = 12.622, *p* = 0.002, η_p_^2^ = 0.375, where the negative effect of Jointness and Congruence on the efficiency of decisions decreased over Time. The Jointness x Symmetry interaction was found significant, *F* (1, 21) = 5.176, *p* = 0.034, η_p_^2^ = 0.198, however, it further interacted with Time, *F* (1, 21) = 8.800, *p* = 0.007, η_p_^2^ = 0.295. To clarify this interaction, the last step of analyses was to test the perspective interference effect in the first and second half of the trials separately in 2 (Jointness) × 2 (Symmetry) repeated measures ANOVA-s.

In the first part of the experiment Symmetry, *F* (1, 21) = 9.489, *p* = 0.006, η_p_^2^ = 0.311 and Congruence, *F* (1, 21) = 87.233, *p* < 0.0001, η_p_^2^ = 0.806, had a main effect on efficiency scores, in addition to a tendency level effect of Jointness, *F* (1, 21) = 3.777, *p* = 0.066, η_p_^2^ = 0.152. Jointness was found to interact with Congruence, *F* (1, 21) = 4.476, *p* = 0.046, η_p_^2^ = 0.176, the deteriorating effect of Jointness was more pronounced on incongruent trials. Crucially, the predicted Jointness x Symmetry interaction was significant, *F* (1, 21) = 8.118, *p* = 0.010, η_p_^2^ = 0.279.

On the second half of the trials, only the significant main effect of Symmetry, F (1, 21) = 28.516, p < 0.0001, η_p_^2^ = 0.576, and of Congruence, *F* (1, 21) = 73.754, *p* < 0.0001, η_p_^2^ = 0.778, was apparent. The Jointness x Symmetry interaction did not emerge, *F* (1, 21) = 0.005, *p* = 0.942, η_p_^2^ = 0.00. No other interactions reached significance, *p*-s > 0.320.

### Discussion of experiment 1

The findings of Experiment 1 show that perspective interference from a task partner’s visual perspective can arise both for symbols that have widely shared and over-practiced meaning and for symbols that the participants learnt within the context of the experiment. To our knowledge, this is the first attempt to test spontaneous perspective taking regarding stimuli that do not have culturally grounded meaning.

However, the spatial structure of the novel asymmetric symbols was different to the structure of asymmetric numbers in an important respect. While the symbols are asymmetric on the horizontal axis only, the numbers are asymmetric on both the horizontal and vertical axes. Therefore, the symbols can be “flipped” while numbers need to be rotated to reach the alternative perspective. Calculating how a 180-degree mirrored object looks from the perspective of someone sitting opposite us is much simpler than having to rotate something to know what the person facing us sees. It may be that the ease of the former computation contributed to the emergence of the spontaneous level-2 interference in the symbol group. To investigate whether the spontaneous level-2 perspective taking would also be observable in a task which requires more cognitive capacity, we ran Experiment 2. In this experiment, we used more complex asymmetric symbols as stimuli, including ones that are asymmetric both horizontally and vertically.

However, during this phase of the investigation, COVID-19 unfortunately struck Hungary. As a result, we could only conduct the study while adhering to strict COVID-19 protocols. Both the researchers and participants wore masks and gloves, and we ensured proper distancing during Experiment 2a. These conditions, though minor, did alter the environment compared to Experiment 1. Therefore, after the COVID-19 situation subsided, we repeated the experiment (Experiment 2b) to ensure that the changes in conditions did not impact our results. Ultimately, the results did not differ, but due to the changes in procedure, we consider it important to describe both of the experiments.

## Experiment 2a

Another set of asymmetric stimuli were created to investigate whether the complexity of the stimuli affect spontaneous level-2 PT or not, and also to better reflect the spatial structure of the numerals 6 and 9 in terms of the rotations needed to ascertain the other person’s perspective.

### Methods

**Participants.** A total of 20 participants were recruited to the experiment in pairs (*M*_*age*_ = 21.4 years, *SD* = 2.68 years, 18 females, 2 males). The participants did not know each other prior to participating in the task.

**Stimuli.** The same symmetric symbols were used as in Experiment 1, while a different pair of asymmetric stimuli were prepared which were asymmetrical on both horizontal and vertical axes to allow variation in complexity (see Fig. 1). New asymmetric symbols were 11.5 cm in height, and 5.2 or 6.2 cm in width similarly to asymmetric symbols used in experiment 1. This was not determined by how easy it was to learn, draw, or recall the symbols. Instead, it was defined by how easy or difficult it may be to recognize them from the perspective of the person sitting opposite. This aspect was especially relevant for asymmetric symbols. In contrast to Experiment 1, this experiment required more effort for the asymmetric symbols, as they were not vertically symmetrical, but required point reflection. To determine if their counterpart saw the same symbol, the participant had to perform an additional mirroring step. They first had to mirror the symbol vertically and then horizontally to align with their counterpart’s view. This extra step in vertical and horizontal mirroring introduced additional complexity. We believe this additional complexity explains the difference in cognitive resources required between Experiment 1 and 2.

**Procedure**. The experimental procedure reflected that of the SV group of Experiment 1, except for the cautionary measures taken in response to the COVID-19 pandemic. The spatial arrangement of the task did not have to be changed compared to the original protocol. Participants sat at a 1.5 m distance during the verification task, in accordance with the social distancing regulations at the time required by Hungary. Furthermore, both the participants and the experimenter wore masks and gloves throughout the experiment.

**Data cleaning.** The data cleaning process was the same as it was in Experiment 1. 4,35% and 4,25% of all data points were identified as outliers in the first and second half of the experiment (total: 4,3%).

### Results

A repeated measures ANOVA with Time, Jointness, Symmetry and Congruence as factors was conducted on all measures.

*Efficiency score.* The main effect of Time, *F* (1, 19) = 41.370, *p* < 0.0001, η_p_^2^ = 0.685, Symmetry *F* (1, 19) = 6.729, *p* = 0.018, η_p_^2^ = 0.262, and Congruence,* F* (1, 19) = 55.652, *p* < 0.0001, η_p_^2^ = 0.745, was found significant. Participants’ decisions were more efficient in the second half of the experiment, for symmetric stimuli and congruent trials. Additionally, there was a significant interaction of Time and Congruence, *F* (1, 19) = 18.833, *p* < 0.0001, η_p_^2^ = 0.498, the difference between congruent and incongruent trials decreased over time. The expected interaction of Jointness and Symmetry was not significant, *F* (1, 19) = 0.047, *p* = 0.831, η_p_^2^ = 0.002. The interaction of Jointness, Symmetry and Congruence approached significance, *F* (1, 19) = 3.370, *p* = 0.069, η_p_^2^ = 0.164, but the expected interaction was not significant in either the congruent, *F* (1, 19) = 2.785, *p* = 0.112, η_p_^2^ = 0.128, or the incongruent trials, *F* (1, 19) = 2.971, *p* = 0.101, η_p_^2^ = 0.135 (in fact, in the congruent trials, the pattern was reversed: larger difference between symmetric and asymmetric stimuli in the individual than the joint block). Thus, the effect of spontaneous level-2 PT could not be replicated with more complex stimuli.

*Reaction Times.* Findings on RT-s closely reflected those on efficiency scores. Time, *F* (1, 19) = 43.737, *p* < 0.0001, η_p_^2^ = 0.697, Symmetry, *F* (1, 19) = 6.046, *p* = 0.024, η_p_^2^ = 0.241, and Congruence,* F* (1, 19) = 80.755, *p* < 0.0001, η_p_^2^ = 0.810, had a main effect on RT-s, participants responding quicker in the second half of the experiment, to symmetric stimuli and congruent trials. Additionally, the interaction of Time and Congruence was significant, *F* (1, 19) = 12.739, *p* = 0.002, η_p_^2^ = 0.401. The Jointness x Symmetry interaction was not present in the data, *F* (1, 19) = 0.391, *p* = 0.539, η_p_^2^ = 0.020. None of the other interactions reached significance.

### Discussion of Experiment 2a

There was no indication of spontaneous level-2 PT in Experiment 2a, during which a more complex set of stimuli was used. This result raises the possibility that the amount of experience with a set of stimuli *in combination with* their spatial complexity and the cognitive load required to process them determines whether the task partner’s alternative perspective interferes with self-perspective based identity judgments.

However, while experiment 2a resembled the SV group in Experiment 1, due to the Covid-19 pandemic, a few changes had to be introduced in the experimental procedure. For instance, participants were wearing gloves and masks throughout the whole process. To avoid the possibility that these circumstances had an influence on the results, Experiment 2b was conducted as a control study Covid-19 pandemic situation ameliorated in the country.

## Experiment 2b

### Methods

**Participants.** 26 adults took part in Experiment 2b and were recruited in pairs. Participants only got to know each other during the experiment.

**Procedure**. The procedure was the same as it was in Experiment 2a, the only difference being that due to the considerable decline of Covid-19 pandemic cases in the country, participants did not wear gloves and masks throughout the experiment. In this way, the circumstances of Experiment 2b were almost identical to that of Experiment 1.

**Data cleaning.** The process of data cleaning reflected that of Experiment 1. 4,53% and 4,17% were excluded as outliers in the first and the second half of the experiment (total: 4,35%).

### Results

A repeated measures ANOVA with Time, Jointness, Symmetry and Congruence as factors was conducted on all measures.

*Efficiency score.* Time, *F* (1, 25) = 39.245, *p* < 0.001, η_p_^2^ = 0.177, Symmetry *F* (1, 25) = 11.051, *p* = 0.003, η_p_^2^ = 0.073, and Congruence,* F* (1, 25) = 56.314, *p* < 0.001, η_p_^2^ = 0.123, had a main effect of efficiency score. Responses were more efficient in the second half of the experiment, for symmetric stimuli and congruent trials. The interaction of Symmetry and Time was found to be significant, *F* (1, 25) = 10.014, *p* = 0.004, η_p_^2^ = 0.004: the difference between symmetric and asymmetric trials decreased over time. The expected interaction of Jointness and Symmetry was not significant, *F* (1, 25) = 0.855, *p* = 0.364, η_p_^2^ = 4.145e-4. According to these results, spontaneous level-2 PT could not be replicated on complex stimuli, not even in the absence of Covid restrictions (e.g. masks and gloves).

*Reaction times.* Findings on RT-s did not notably differ from those on efficiency scores. The main effect of Time* F* (1, 25) = 55,680, *p* < 0.001, η_p_^2^ = 0.244, Symmetry, *F* (1, 25) = 14.499, *p* <  = 0.001, η_p_^2^ = 0.050, and Congruence,* F* (1, 25) = 165.967, *p* < 0.001, η_p_^2^ = 0.168, was found to be significant. Participants made quicker decisions in the second half of the experiment, in case of symmetric stimuli and congruent trials. Additionally, the interaction of Symmetry and Time *F* (1, 25) = 11.499, *p* = 0.002, η_p_^2^ = 0.002 was significant. The expected interaction of Jointness and Symmetry was not found to be significant *F* (1, 25) = 2.950e-5, *p* = 0.996, η_p_^2^ = 1.348e-8. Our findings revealed no L2PT effect triggered by complex newly learnt symbols.

## General discussion

Whether humans engage in spontaneous, online L2PT has been vigorously investigated recently. With a few exceptions, our knowledge about this capacity is obtained through paradigms that require participants to make identity judgments regarding perspective-dependent and perspective-independent alphanumeric symbols (mostly, Arabic numbers). In contrast, a real or computerised other agent occupies an opposite perspective. Arabic numbers in Western cultures have two features that may potentially be relevant when representing others’ perspectives on them: the ease with which their meaning is identified, recalled from long-term memory, and the expectation that given the same spatial perspective, other people will identify and interpret the symbol the same way participants themselves do.

The study presented here addresses the role of the first characteristic, namely, extensive background knowledge. In Experiment 1, we compared the performance of two groups of participants, one that performed the verification task on Arabic numbers and one that did the same on newly acquired symbol-label pairs. In addition to being less automated, the processing of novel symbols is also free from the pervasive effects of the number domain (e.g. numerical distance effect, Moyer and Landauer 1967, representation of zero, Nieder 2016, or spatial relations, Wood et al. 2008) which may influence decisions in the number verification task in uncontrolled ways.

Our investigations demonstrated that while participants’ decisions remained less efficient (less automatized) for newly learnt symbols, their self-perspective-based decisions were, nevertheless, influenced by their partner’s conflicting perspective, when the symbols were asymmetric on one axis in Experiment 1. This finding corroborates previous findings with 8–9.5-year-old children showing an adult-like L2PT effect despite lower familiarity with alphanumeric characters and higher task demands on children (Elekes et al. 2017).

However, when we investigated the possibility that the amount of the cognitive load may play an important role in the process of level-2 PT,—by changing the novel symbols being asymmetric on both axes in Experiment 2a and 2b—we found that the spontaneous level-2 PT effect diminished. According to these results, the online PT mechanism does not require proficiency in identifying the specific symbols, but the complexity and the presumably applied cognitive load may influence the process. The results of Experiment 2 suggest that the cognitive capacity loaded by the processes of learning, retrieving, and computing the other’s perspective of recently learnt symbols can surpass the capacities of the online PT mechanism.

The procedure we employed ensured that participant pairs had established a common ground about the symbol-label pairs before starting the verification task: they recreated the pairs jointly and formed a consensus explicitly. Therefore, the expectation of shared knowledge that characterises people’s assumptions in case of symbols with culturally grounded meaning was maintained in the experiment, though in a more constrained way, potentially only applicable to the experimental context and the given dyad of participants. Consequently, whether sharing knowledge about the stimuli is a prerequisite of altercentric interference remains unanswered.

This issue relates to a core question about the online perspective taking paradigms we commonly use: whether they tap into a process that computes in what *subjective* way a fellow individual mentally represents a specific piece of information. Perspective taking proper (representing the aspect under which a certain object is represented by someone, Perner et al. 2003) should be sensitive to the individual characteristics of the partner’s mind that give rise to those subjective representations. Importantly, a difference in perspectives can not only come from a difference in spatial positions, but also from differences in background knowledge (e.g. an object that is encoded as a rock to one person may be encoded as a sponge by another who has received information about its substance, Sprung et al. 2007). Similarly, a symbol that has alternative meanings from different viewing angles will only convey different meanings to the partner if they have the competence to infer that meaning from appearance (even if meaning, in this case, is constrained to a pertaining label). Therefore, if online PT tracks how certain objects are *interpreted* by someone as opposed to how it is visually represented for them (as argued also by Cole and Millet, 2019, but contrary to views of Philips, 2019), it should be selective based on the partner’s assumed knowledge about the visual stimulus.

There is some indication from developmental psychology that perspective taking is sensitive to the partner’s knowledge about the stimuli: when 4-year-olds are asked to tell what number is on the Table (6/9), they rely on their own viewpoint if prior information indicates that the partner knows Arabic numbers, but take the partner’s view when she is uncertain about the numbers and wants to learn about them (Zhao et al. 2016b). Findings in online tasks are mixed in this respect. Elek and colleagues (Elek et al. 2019) report that 9-year-old children show online L2PT selectively for ingroup partners over outgroup partners when groups are defined based on linguistic cues of cultural group-membership. Also, Elekes et al. (2016) found perspective interference in adults only when the partner had to attend to and represent the symbol’s meaning (over presentation colour). On the other hand, Surtees et al. (2016a) found perspective interference regardless of the partner’s task and the consequent representation of the object, and there is even indication of PT-like effects when the partner’s gaze is averted from the numbers (Kuhn et al. 2018; Ward et al. 2019a, b). As prior information about the symbol-label pairs is under full control in the SV version of the online L2PT task, this provides a fruitful avenue to further test the role that the partner’s knowledge about the stimuli plays in the occurrence of the interference effect.

Although in the form of an incidental finding and thus in a speculative manner, the present set of data might also speak to this matter. The sole difference in findings between the NV and SV groups in Experiment 1 was that the altercentric interference effect deteriorated from the first to the second half of the experiment in the SV group, while it remained unaffected by time in the NV group, in which participants made identity ssssjudgements about familiar numbers. A possible explanation relates to the nature of common ground that participants may have had regarding each type of stimuli. Common ground is not a unitary construct. For instance, Kecskés and Zhang (2009) propose that human interactions are guided by an a priori common ground (lasting expectations that individuals enter the interaction with) and an emergent common ground that is obtained and changes dynamically in the course of the interaction. Analogously, the common ground regarding numbers and novel symbols in our experiments likely differed in their temporal validity. The meaning of numbers constitutes lasting and broadly shared consensus that does not originate in the experimental context. On the contrary, participants’ shared expectation about the symbol-label pairs was created within the experiment and were thus a more fragile and temporal construct that may have faded off as time passed. If perspective taking indeed functions on the level of subjective interpretations, the diminishing common ground might lead to the deterioration of the online PT effect in the SV group. Another alternative explanation could be that the effect diminished due to cognitive overload. Continuous mental effort is required to learn, identify, and recall novel symbols. Therefore, it is conceivable that as participants became more tired, it may have become more difficult for them to monitor the other person’s perspective. This aspect of our data and potential theoretical implications need further exploration.

It is also important to emphasise that although the other person’s attention could potentially have acted as a confounding variable in the experiment, we do not believe that this was the case. If mutual attention were a confounding factor, we would expect it to affect performance similarly for both symmetric and asymmetric symbols in the individual and joint conditions. However, significant differences between these conditions were only found for asymmetric symbols. Furthermore we did not observe significant differences in Experiment 2 either. Although we acknowledge that external attention may have played some role, our results suggest that it was not a confounding variable in our research. Future studies could investigate the influence of external attention in more detail by modifying the paradigm and changing participants’ visual access or environmental conditions (e.g., by having participants sit next to each other or wearing opaque or regular sunglasses).

To summarise, the aim of the current study was to take a step beyond prior work on online L2PT. It assessed participants’ responses to newly learnt symbol-label pairs compared to highly familiar, culturally grounded alphanumeric characters. This empirical investigation aimed to provide an important addition to our knowledge of online L2PT by untangling whether previous findings are generalizable to new stimuli. We found evidence for altercentric interference after a relatively short training phase on the novel symbols. This result indicates that the PT effect is not constrained to objects that participants can identify automatically. On the other hand, the results also demonstrated that the complexity of the recently learnt symbols had an impact on the process of spontaneous level-2 PT. It means that the cognitive capacity involved in the process may have an important role. Altercentric interference appears in the case of familiar and well-known stimuli (eg. numbers) that are identified automatically without any cognitive effort. It is also observable but more fragile (disappeared in the second half of the experiment) with recently learnt symbols that require moderate cognitive exertion (Experiment 1), and it fades away if the new symbols are more complex (Experiment 2). These results suggest that the spontaneous L2PT process may work in function of the available cognitive resources, however, further studies should investigate this question in more detail.

## Footnotes

1 Whether people encode object locations (left/right) relative to their own spatial perspective or another agent’s is sometimes also referred to as level-2 PT, but falls outside of our focus (Furlanetto, Cavallo, Manera, Tversky, & Becchio 2013; Tversky & Hard 2009).
